# Screening of ferroptosis-related genes with prognostic effect in colorectal cancer by bioinformatic analysis

**DOI:** 10.3389/fmolb.2022.979854

**Published:** 2022-09-20

**Authors:** Dongzhi Hu, Zhengyang Zhou, Junyi Wang, Kegan Zhu

**Affiliations:** Key Laboratory of Cancer Prevention and Therapy, National Clinical Research Center for Cancer, Tianjin’s Clinical Research Center for Cancer, Tianjin Medical University Cancer Institute and Hospital, Tianjin Medical University, Tianjin, China

**Keywords:** colorectal cancer, ferroptosis, prognostic model, immune microenvironment, bioinformatic analysis

## Abstract

Colorectal cancer (CRC) remains a common malignant tumor of digestive tract with high incidence rate and high mortality in the worldwide. The current clinical treatments of CRC often fail to achieve satisfactory results. Searching for more effective prediction or prognosis biomarkers, or developing more targeted therapeutic schedule may help to improve the outcomes of CRC patients. Here, we tried to study the effect of ferroptosis-related genes on CRC prognosis and make it clearer that ferroptosis has connection with immune environment. First, we obtained gene expression data of CRC and normal tissues, as well as corresponding clinical data from the Gene Expression Omnibus (GEO) database and the Cancer Genome Atlas (TCGA) database. The differentially expressed genes (DEGs) were intersected with ferroptosis-related gene set downloaded from FerrDb database, and 93 abnormally expressed ferroptosis-related genes were obtained. Then, these genes were analyzed for functional enrichment. Univariate Cox regression and multivariate Cox regression analyses were performed to establish prognostic model based on ferroptosis-related genes. In the process of exploring the correlation between prognostic genes and immune infiltration, we found that these genes were closely related to B cells, CD8^+^ T cells, CD4^+^ T cells, macrophages and other cells in CRC. In addition, we found a large proportion of plasma cells and macrophages in TCGA-COADREAD. Finally, a prognostic nomogram of ferroptosis-related genes was established, including age, sex, grade and other predicted values. To summary, we established a prognostic model of colorectal cancer (CRC) based on ferroptosis-related genes and further explored the relationship between these genes with immune microenvironment.

## Introduction

According to statistics in 2021, colorectal cancer (CRC) ranks third in morbidity and second in mortality worldwide, seriously affects human health and brings heavy economic burden (Li et al., 2021; Rezapour et al., 2021; Sung et al., 2021). Especially in China, CRC is one of the most common malignancies after lung cancer, with increasing morbidity and mortality (Arnold et al., 2017; Zhou et al., 2021). Usually, the CRC patients only show symptoms at the advanced stage, which make it difficult in the early detection. In fact, many patients are already in advanced cancer when first diagnosed (Simon 2016; Dekker et al., 2019; Biller and Schrag 2021). Besides, no regular physical examination due to economic reasons or lack of awareness is also an important reason for the late diagnosis of CRC (Chen et al., 2019; Ladabaum et al., 2020). Patients with advanced CRC have poorer prognosis, whose five-year survival rate is only 10% (Brenner et al., 2014; Dekker et al., 2019). The common clinical treatments, including surgery, chemotherapy, and immunotherapy, all failed to achieve satisfactory results (Piawah and Venook 2019; Johdi and Sukor 2020; Biller and Schrag 2021). Therefore, it is urgent to screen for more effective biomarkers for early diagnosis and prognosis, or develop more potential therapeutic targets for CRC, as well as other tumors (Mohamed et al., 2020).

Ferroptosis, which was first proposed in 2012, is a non-apoptotic and iron-dependent form of cell death characterized by the accumulation of reactive oxygen species (ROS) (Dixon et al., 2012). It is significantly different from apoptosis, necrosis and autophagy in both cell morphology and cellular function, which are also important bases to distinguish them (Xie et al., 2016; Li et al., 2020). In recent years, there has been an increasing number of studies on ferroptosis. It has been reported that ferroptosis can be triggered by different physiological conditions or pathological stress (Mou et al., 2019; Jiang and Stockwell 2021). More and more evidences show that ferroptosis has a regulatory effect on the occurrence and development of many diseases (Dixon et al., 2012; Fearnhead and Vandenabeele 2017; Jiang and Stockwell 2021; Wu et al., 2021). It should be noted that ferroptosis also plays important roles in different cancers (Liang and Zhang 2019; Koppula et al., 2021). Moreover, the role of ferroptosis in immune microenvironment has attracted more and more attention recently (Stockwell and Jiang 2019; Wang et al., 2019; Chen and Kang 2021; Lu et al., 2021). Tumor microenvironment contains a large number of immune cells which act in chemotherapy and antiangiogenic therapy of CRC, involving immune tolerance, immune escape and other processes (Wang et al., 2019). Tumor microenvironment may be a crucial bridge by which ferroptosis functions in cancers. Thus, a more in-depth study of the relationship between ferroptosis and immune cell infiltration may open a new way for immunotherapy of CRC.

Previous studies on ferroptosis in tumors mainly focused on the abnormally expressed ferroptosis-related genes, which have close connections with tumors, by screening the relative database through bioinformatic analysis. Here, we want to further analyse ferroptosis-related factors that have connections with immune microenvironment. In our study, CRC expression data together with corresponding patients’ information from TCGA and GEO databases were screened and cross-referenced with FerrDB database to identify differentially expressed genes (DEGs) associated with ferroptosis. The prognostic genes were further screened and prognostic model was established to predict the prognosis of CRC patients. Meanwhile, we analyzed the association between ferroptosis-related genes and tumor microenvironment in CRC, enhancing our understanding of the relationship between ferroptosis and immune cell abundance. In a word, our results provide a novel ferroptosis-related model for prognosis analysis of CRC patients and further verified the relationship between ferroptosis and immune microenvironment, which may contribute to the immunotherapy in the future.

## Materials and methods

### Data source

From the GEO database (https://www.ncbi.nlm.nih.gov/geo), we downloaded RNA expression data including normal and tumor tissues from GEO: GSE21510, GSE44861, GSE62321 and GSE79793, and obtained RNA expression data as well as clinical information of patients from GEO: GSE41258. All the above data are normalized by log_2_-scale transformation to ensure standardization. The gene symbols with multiple probes were calculated using mean expression levels. We also obtained the level three HTSEQ-FPKM format RNA sequencing data in CRC project from TCGA database (https://www.cancer.gov/tcga/), named TCGA-COADREAD. A total of 644 matched patients’ clinical information and sample information were obtained. In addition, a total of 388 ferroptosis-related genes (including drivers, markers, and suppressors) were obtained from FerrDb database (http://www.zhounan.org/ferrdb/legacy/index.html) as candidate genes. Detailed information about these genes are shown in Supplementary Table S1. This study followed the publication guidelines of the GEO and TCGA databases.

### Identification of differentially expressed genes (DEGs)

We used R package “limma” in RStuio to detect the DEGs between tumor and normal tissue from GEO: GSE21510, GSE44861, GSE52321, GSE79793 with *p*-value < 0.05 and |log2FC| ≥1. R package “PheatMap” was applied to visualize the degree rang of differences between the four datasets. Next, we obtained 93 ferroptosis-related genes by intersecting DEGs and candidate genes.

### Functional analysis of ferroptosis-related genes

The GO and Kyoto Encyclopedia of Genes (KEGG) analysis were performed using a gene annotation and analysis resource Metascape (https://metascape.org/gp/index.html#/main/step1). The cutoff of the *p*-value was 0.01. Enriched terms were selected to construct the network, and similar terms were connected with edges. The cutoff value of similarity is 0.3. STRING (http://string.embl.de/) was used to predict PPI information. Then, we established PPI networks using Cytoscape. And the MCODE algorithm was performed to identify the key modules. The cutoff of the *p*-value was 0.05.

### Construction and validation of prognostic models

We used TCGA-COADREAD (as the training cohort) and GEO: GSE41258 datasets (as the validation cohort) to establish prognostic markers of ferroptosis-related genes. Univariate Cox analysis of OS was conducted to identify ferroptosis-related genes with significant prognostic value, and *p*-value < 0.05 was considered to be statistically significant. The independent prognostic factors were identified by multivariate Cox regression analysis. The prognostic model of ferroptosis-related genes was constructed according to the correlation coefficient of independent prognostic genes. Patients in the TCGA-COADREAD were divided into low-risk group and high-risk group according to the risk scoring algorithm obtained by multivariate Cox regression analysis, and the survival curve was drawn. The ROC curve was plotted using R package “time ROC”, and the prognostic efficiency was evaluated according to AUC.

### Immune analysis of ferroptosis-related prognostic genes

To determining the immune correlation of prognostic genes, we used TIMER2.0 database (http://timer.cistrome.org/) to analyze the relationship between prognostic genes and tumor immune infiltrating cells. Then, the R package “estimate” was used to calculate the immune score of TCGA-COADREAD to obtain the stromal score, immune score, and ESTIMATE score of prognostic genes. And a Kaplan-Meier survival curve was plotted to evaluate the relationship between immune score and patient survival time. CIBERSORTx (https://cibersortx.stanford.edu/) was used to evaluate the proportion of immune cell infiltration in tissues from TCGA-COADREAD and GEO: GSE41258 datasets to clarify the relationship between prognostic genes and immune cell infiltration. In addition, we also used ssGSEA algorithm to calculate the distribution of various immune cells and draw violin plots.

### Construction of a nomogram

We used R package “rms” to plot the nomogram and calibration curves. A nomogram could provide survival probability for a specific outcome, and calibration curve (3-years OS) was used to visualize the observed rates against nomogram-predicted probabilities.

## Results

### Identification of DEGs associated with ferroptosis in CRC

**FIGURE 1:**
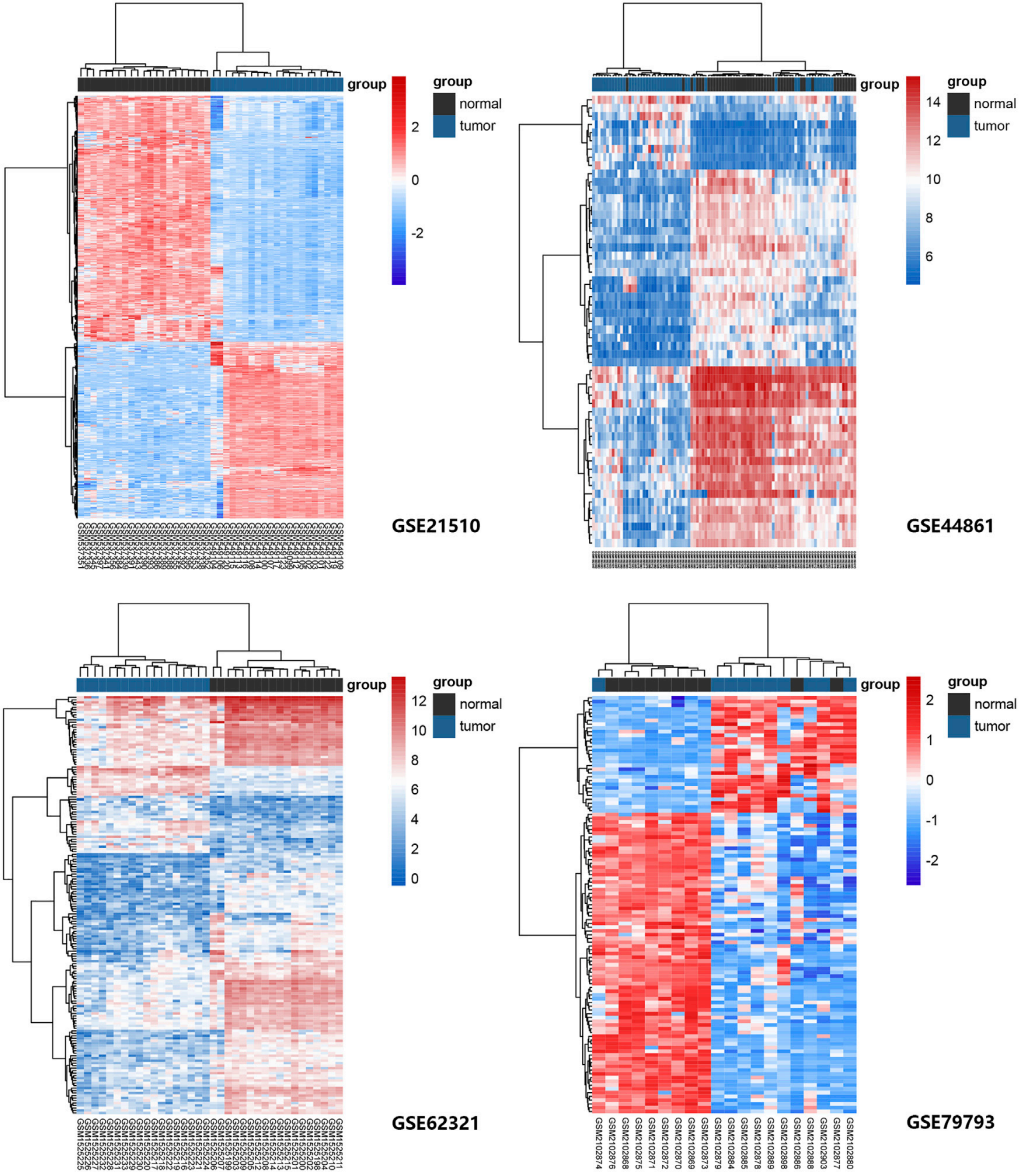
Heat maps of ferroptosis-related gene expression in normal and tumor samples in GEO: GSE21510, GSE44861, GSE62321, and GSE79793 datasets. The genes were clustered according to their expression levels. The red color represents high expression and the blue color represents low expression.

**FIGURE 2:**
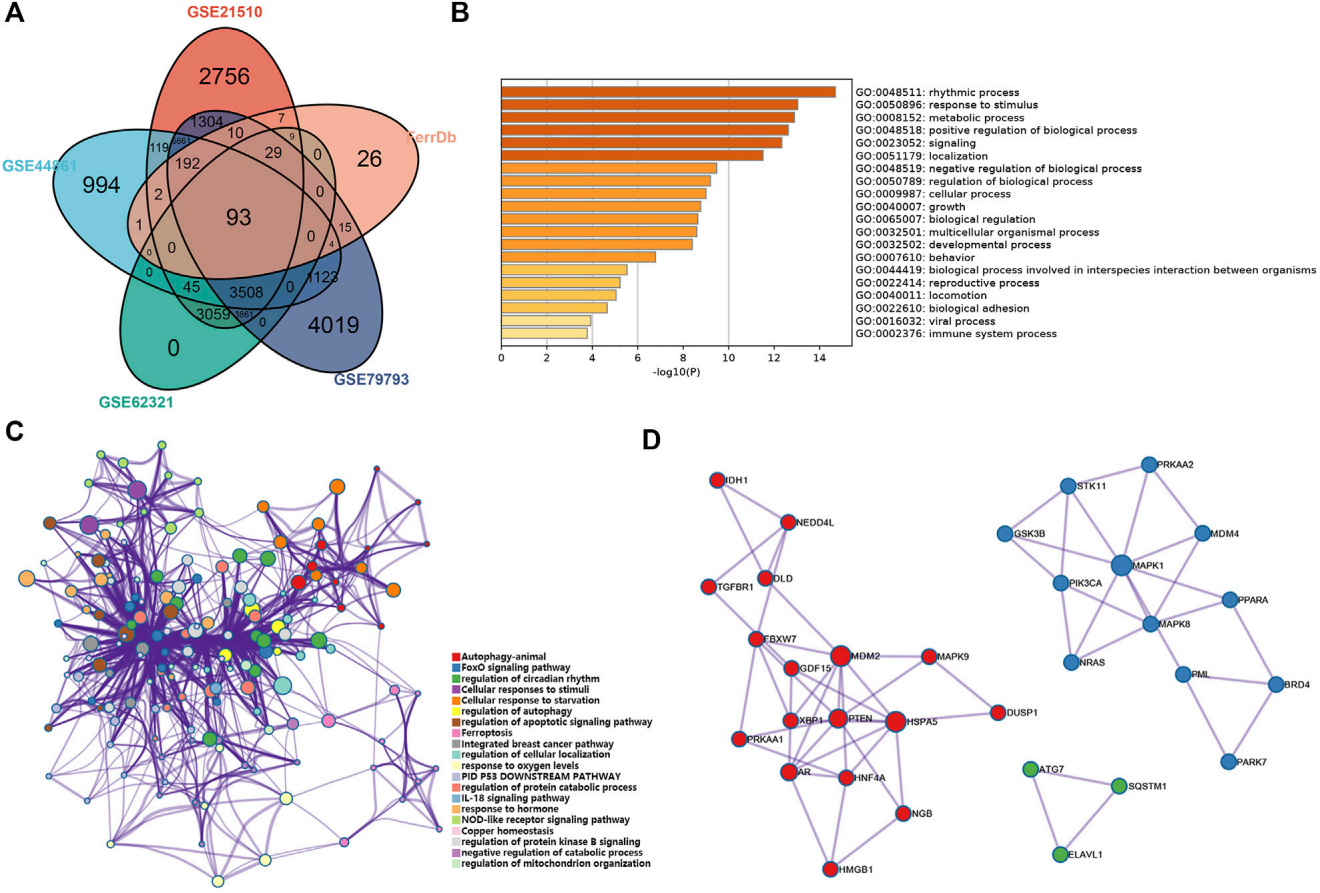
Analysis of the ferroptosis-related DEGs in CRC. **(A)** Venn diagram of the abnormally expressed ferroptosis-related genes that common to the four datasets (GEO: GSE 21510, GSE44861, GSE62321, and GSE79793) and FerrDb database. **(B,C)** Bar plot (enriched *p*-value) and network (enriched terms cluster ID) showed the distribution and relationships of various functions based on the GO and KEGG analysis by Metascape. **(D)** Three MCODE identified in the PPI network displayed the ferroptosis-related hub genes.

Four GEO datasets were selected as data sources, and the information is listed in Table 1. Through the differential gene analysis, a total of 2,611 up-regulated genes (log_2_FC > 1, *p*-value < 0.05) and 471 down-regulated genes (log_2_FC < 1, *p*-value < 0.05) were obtained from GEO: GSE21510. A total of 141 genes showed upregulation and 261 genes showed downregulation were obtained from GEO: GSE44861. In addition, 254 genes with high expression and 715 genes with low expression were found in GEO: GSE62321, 46 genes with high expression and 241 genes with low expression were found in GEO: GSE79793. The heat maps of the four datasets are showed in Figure 1. The DEGs associated with ferroptosis were obtained by intermixing these DEGs with identified ferroptosis-related genes from FerrDb database. The Venn diagram displayed that there are 93 genes intersecting between five datasets (Figure 2A). All genes are listed in Supplementary Table S1.

**TABLE 1:** The information of datasets from the GEO database.

Accession number	Platform	Samples	Experiment type	PMID
GEO:GSE21510	GPL570	46	Expression profiling by array	21,270,110
GEO:GSE 44861	GPL3921	94	Expression profiling by array	23,982,929
GEO:GSE62321	GPL97	30	Expression profiling by array	24,023,955
GEO:GSE79793	GPL14951	20	Expression profiling by array	28,595,259

We performed functional analysis by Metascape to investigate the underlying mechanisms of abnormally expressed ferroptosis-related genes in CRC. The gene ontology (GO) analysis results revealed that the dysregulated ferroptosis-related genes were mainly enriched in response to stimulus, metabolic process and positive regulation of biological process (Figures 2B,C). It is worth noting that immune system process was also enriched. It suggested that there may be a certain correlation between ferroptosis-gene set and tumor immune environment. In addition, we used the protein-protein interaction (PPI) network and Molecular Complex Detection (MCODE) plugin based on the Metascape to identify the significant modules in these ferroptosis-related genes. Module 1 involved PTEN, MDM2, HSPA5, AR, HNF4A, and MAPK9. Module 2 involved MAPK1, MDM4, GSK3B, STK11, MAPK8, PIK3CA, and PRKAA2. Module 3 involved SQTM1, ATG7, and ELAVL1 (Figure 2D). Besides, GeneMANIA database was used to verify the interaction of the relative proteins (Supplementary Figure S1A). We also analyzed the transcription factors of these ferroptosis-related genes using PASTAA and presented the top 30 genes in Table 2.

**TABLE 2:** The top 30 transcription factor of 93 ferroptosis-related genes from PASTAA.

Rank	Matrix	Transcription Factor	Association Score	*p*-Value
1	NFY_01	N/A	5.270	1.61e-04
2	MAZR_01	Mazr	4.116	1.90e-03
3	ATF4_Q2	Atf-4, Atf4	3.869	3.19e-03
4	TAXCREB_02	Creb, Deltacreb	3.580	5.71e-03
5	NRL_HAND	N/A	3.560	5.83e-03
6	NFY_Q6_01	Cbf-a, Cbf-b	3.559	5.86e-03
7	MMEF2_Q6	N/A	3.487	6.76e-03
8	IRF1_01	Irf-1	3.148	1.33e-02
9	NFY_Q6	Cbf-a, Cbf-b	3.131	1.37e-02
10	ARP1_01	Coup-tf2	3.076	1.49e-02
11	MTATA_B	N/A	3.042	1.64e-02
12	CRX_Q4	Crx, Rx	2.978	1.95e-02
13	FAC1_01	Fac1	2.940	2.05e-02
14	CREBP1CJUN_01	Atf-2, C-jun	2.877	2.29e-02
15	AMEF2_Q6	Amef-2	2.823	2.52e-02
16	RFX1_02	Rfx1	2.816	2.57e-02
17	PBX1_03	N/A	2.802	2.65e-02
18	LYF1_01	N/A	2.718	3.15e-02
19	PITX2_Q2	Pitx2, Pitx2	2.516	4.50e-02
20	SREBP1_01	Srebp-1, Srebp-1a	2.509	4.55e-02
21	FOX_Q2	Foxd3, Foxf1	2.474	4.88e-02
22	BEL1_B	N/A	2.467	4.96e-02
23	MAF_Q6	N/A	2.380	5.81e-02
24	SRY_02	Sry	2.369	5.93e-02
25	CAAT_C	N/A	2.306	6.70e-02
26	CHOP_01	C/ebp, C/ebpalpha	2.283	6.88e-02
27	STAF_01	Staf	2.252	7.05e-02
28	ATF3_Q6	Atf3	2.241	7.52e-02
29	ATF_B	N/A	2.241	7.52e-02
30	LUN1_HAND	N/A	2.238	7.52e-02

### The establishment and verification of a prognostic model

**FIGURE 3:**
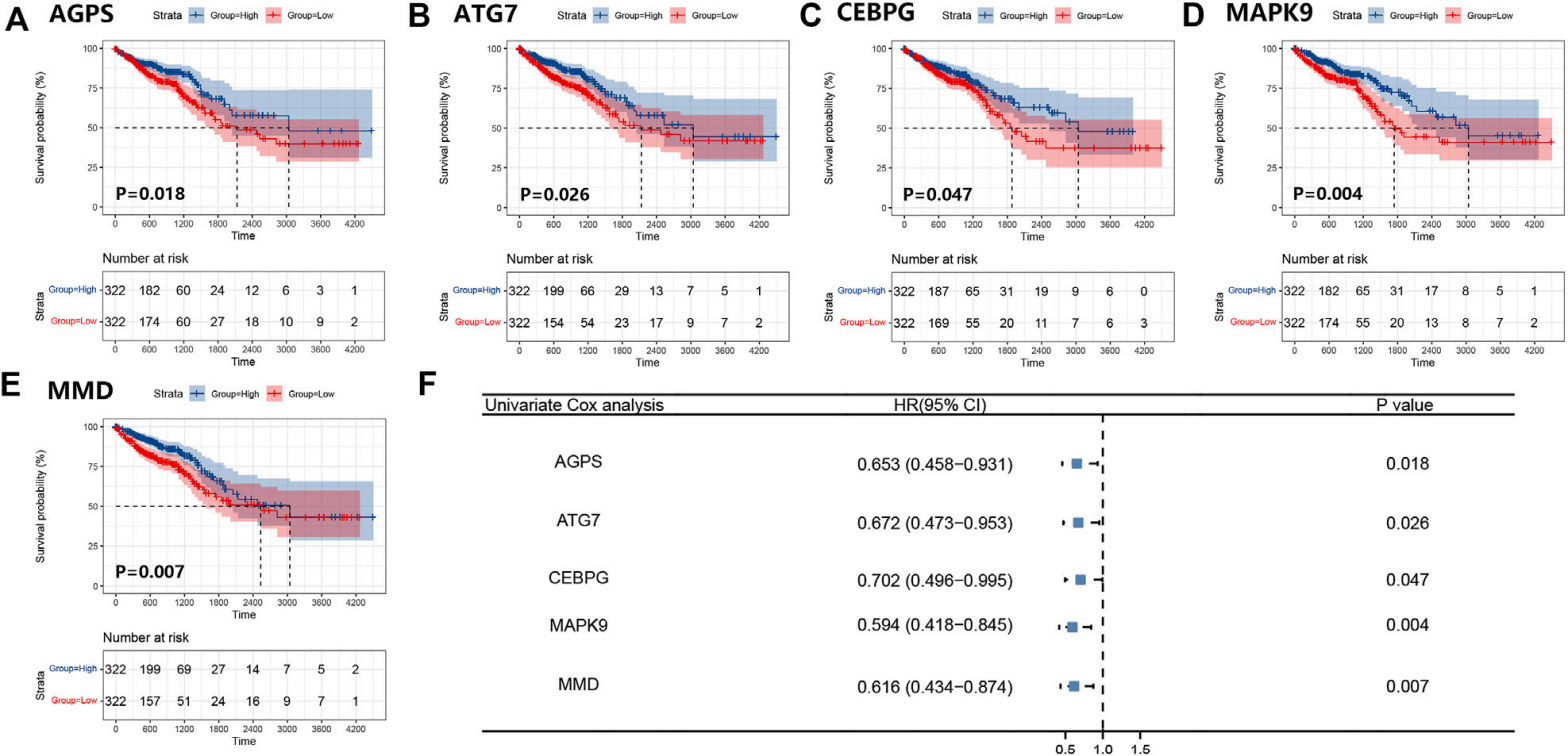
Survival curves of five ferroptosis-related prognostic genes and the forest map of univariate Cox regression analysis. **(A–E)** Kaplan-Meier plots showed the ferroptosis-related genes AGPS **(A)**, ATG7 **(B)**, CEBPG **(C)**, MAPK9 **(D)**, and MMD **(E)** with prognostic value. **(F)** The result of the univariate Cox regression analysis was presented by forest map.

**FIGURE 4:**
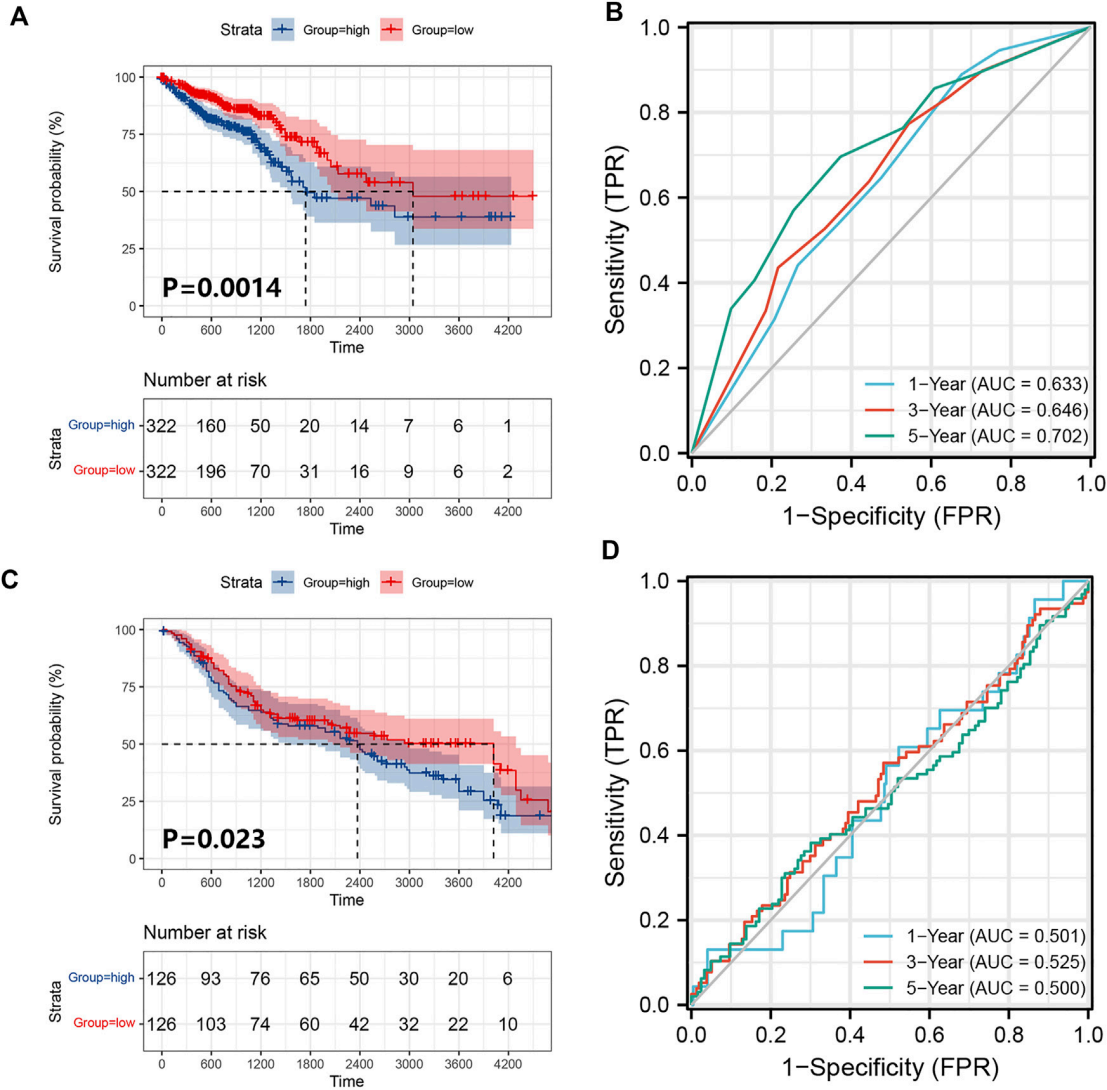
Establishment and verification of the prognostic model. **(A)** A prognostic model for patients with CRC based on the risk coefficients of ferroptosis-related genes ATG7, MAPK9 and MMD in TCGA-COADREAD. **(B)** The ROC curve of the three ferroptosis-related genes with the AUC in TCGA-COADREAD. **(C)** A prognostic model was established from three ferroptosis-related genes in the GEO: GSE41258. **(D)** The ROC curve of the three ferroptosis-related genes with the AUC in GEO: GSE41258.

We obtained 644 standardized mRNA expression data and corresponding patients’ information from TCGA-COADREAD, which were used to establish a predictive model based on ferroptosis-related genes. To improve the accuracy and reliability of the predictive model, GEO: GSE41258 from GEO was used as a validation cohort. Firstly, univariate Cox regression analysis was performed to detect genes significantly associated with prognosis. As shown in Figures 3A–E, five ferroptosis-related prognostic genes were identified from 93 screened genes, which were AGPS, ATG7, CEBPG, MAPK9, and MMD. Figure 3F displayed the forest map of univariate Cox regression analysis. The detailed information of these genes is showed in Table 2. By multivariate Cox regression analysis, ATG7, MAPK9, and MMD were identified as independent prognostic genes (Table 4). Then, we established a prognostic model based on multivariate Cox regression. A risk score for each patient was calculated as follows: (-0.397,447) (β1) × (expression of ATG7) + (-0.575,347) (β2) × (expression of MAPK9) + (-0.385,768) (β3)× (expression of MMD). Then, a high-risk group (n = 322) and a low-risk group (n = 322) were stratified based on the median of the risk score and survival curves were plotted. It was found that the survival time of patients in the low-risk group was significantly longer than that in the high-risk group (Figure 4A). Next, a receiver operating characteristic (ROC) curve was created to assess the prognosis prediction efficiency of the model. As shown in Figure 4B, we found that the area under curve (AUC) was 0.64 (1-year OS), 0.64 (3-years OS), and 0.71 (5-years OS) respectively, which suggested that the predictive effect of the model was acceptable. Furthermore, to evaluate the accuracy and reliability of this predictive model, we validated the power of the model in GEO: GSE41258. In Figure 4C, Kaplan-Meier plots demonstrated that the ferroptosis-related predictive model successfully stratified CRC patients into the long-term OS and short-term OS group with significant difference (*p* = 0.023). Similarly, the ROC curve indicated that the model had an ideal prediction effect (Figure 4D).

### Analysis of prognostic genes in CRC patients

Subsequently, we further analyzed these three prognostic genes, ATG7, MAPK9 and MMD. Table 3 shows that the three genes all belong to ferroptosis driver genes, which obviously have the function of promoting ferroptosis. As shown in Supplementary Figure S1B, the expression levels of ATG7 and MAPK9 were higher in CRC tissues than in normal tissues. The expression of MMD in normal tissues was higher than that in CRC tissues. In addition, we found that the expression of these genes decreased with tumor progression (Supplementary Figure S1C), but it was not statistically significant.

**TABLE 3:** The information of 10 prognostic genes.

Genes	Name	Ferroptosis Property	HGNC ID
AGPS	alkylglycerone phosphate synthase	Driver	327
ATG7	Autophagy related 7	Driver	16,935
CEBPG	CCAAT enhancer binding protein gamma	Marker	1837
MAPK9	Mitogen-activated protein kinase 9	Driver	6,886
MMD	monocyte to macrophage differentiation associated	Driver	7,153

**TABLE 4:** Multivariate Cox regression analysis of signature in TCGA-COADREAD cohort.

Variable	Coef	Exp (coef)	Se (coef)	Z	*p* value
ATG7	−0.397447	0.672033	0.185670	−2.141	0.03231
MAPK9	−0.575347	0.562510	0.193735	−2.970	0.00298
MMD	−0.385768	0.679928	0.190712	−2.023	0.04310

**FIGURE 5:**
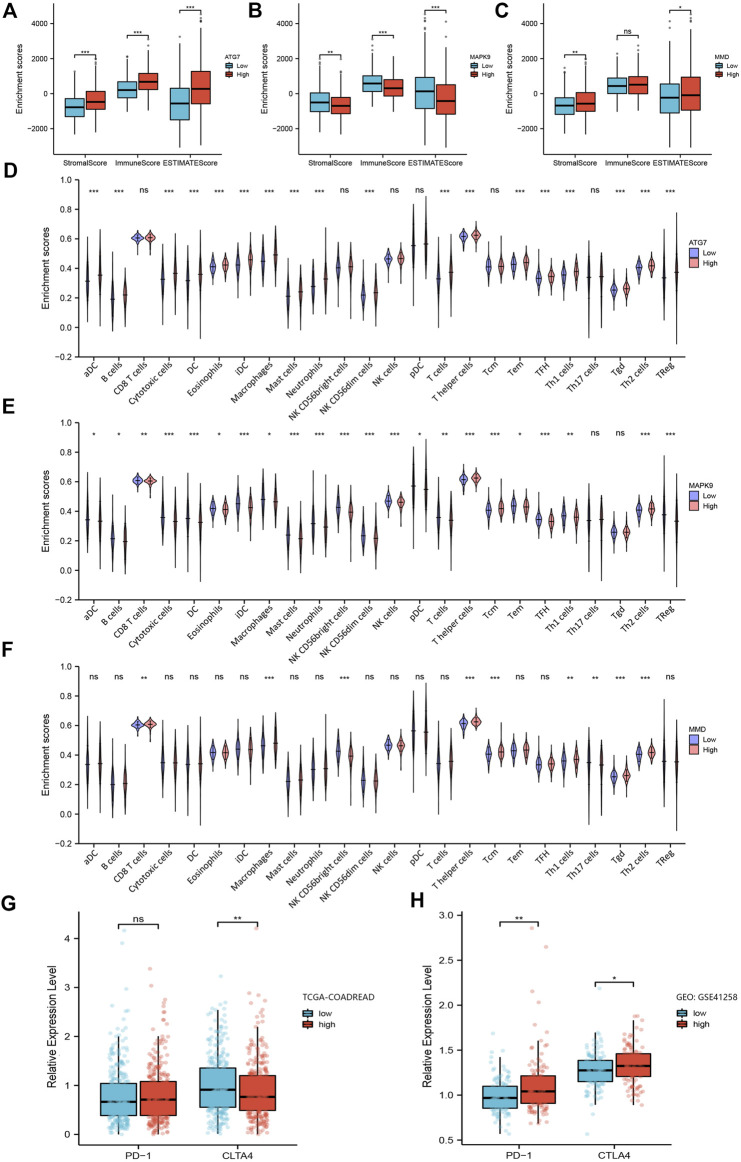
Analysis of the relationship between ferroptosis-related prognosis genes with immune microenvironment. **(A–C)** Box plots of ferroptosis-related genes ATG7, MAPK9 and MMD obtained by the ESTIMATE algorithm, showing their stromal score, immune score and ESTIMATE score. **(D–F)** Violin plots of ferroptosis-related genes ATG7, MAPK9 and MMD obtained by ssGSEA algorithm, showing the distribution of various immune cells between low group and high group. **(G–H)** The expression level of PD-1 and CTLA4 in the low-risk group and high-risk group in TCGA-COADREAD cohort and GEO: GSE41258, respectively. *, *p* < 0.05; **, *p* < 0.01; ***, *p* < 0.001.

In the functional enrichment analysis, we got hints that these genes were involved in immune system processes. Therefore, it is necessary to further explore their relevance with immune infiltration. First, by using the tumor immune estimation resource (TIMER) 2.0 database, we found that these three prognostic genes were associated with B cells, CD8^+^ T cells, CD4^+^ T cells, macrophages, neutrophils, and myeloid dendritic cells in CRC (Supplementary Figure S2A-C4). Next, we calculated the stromal score, immune score, and ESTIMATE score for 644 patients in the TCGA-COADREAD through the ESTIMATE algorithm. The results showed that except the immune score of MMD, the other groups could be statistically divided into low group and high group (Figures 5A–C). Furthermore, Kaplan-Meier plots were performed based on the three scores, but there were no statistical significance between the low stromal/immune/ESTIMATE groups and high stromal/immune/ESTIMATE groups.

Next, we assessed the proportion of immune landscapes in the tumor. Based on the data of gene expression, CIBERSORT algorithm was used to explore the proportion of immune cells in TCGA-COADREAD and GEO: GSE41258 datasets. It was found that plasma cells and macrophages (including the M0, M1 and M2 subsets) accounted for a large proportion of infiltrating immune cells in TCGA-COADREAD (Supplementary Figure S3). We used the ssGSEA algorithm to draw the violin plots for visualizing the distribution of various immune cells between the low group and high group. And we found that the proportions of B cells, CD8^+^ T cells, dendritic cells and macrophages were statistically significant (Figures 5D–F). In addition, M0, M1, M2 macrophages, and plasma cells accounted for a large proportion of infiltrating immune cells in GEO: GSE41258, which was as same as the trend we found in TCGA-COADREAD (Supplementary Figure S4).

Furthermore, we explored the expression of PD-1 and CTLA4 in the low-risk group and high-risk group. It was demonstrated that patients in the high-risk group had a higher expression level of PD-1 and a lower expression level of CTLA-4 in TCGA-COADREAD cohort (Figure 5G). But we noticed that the expression of PD-1 showed no statistical significance between the low-risk group and high-risk group. Additionally, PD-1 in GEO: GSE41258 exhibited consistency with TCGA-COADREAD, while CLTA4 exhibited the opposite trend (Figure 5H).

### Construction of the nomogram

**FIGURE 6:**
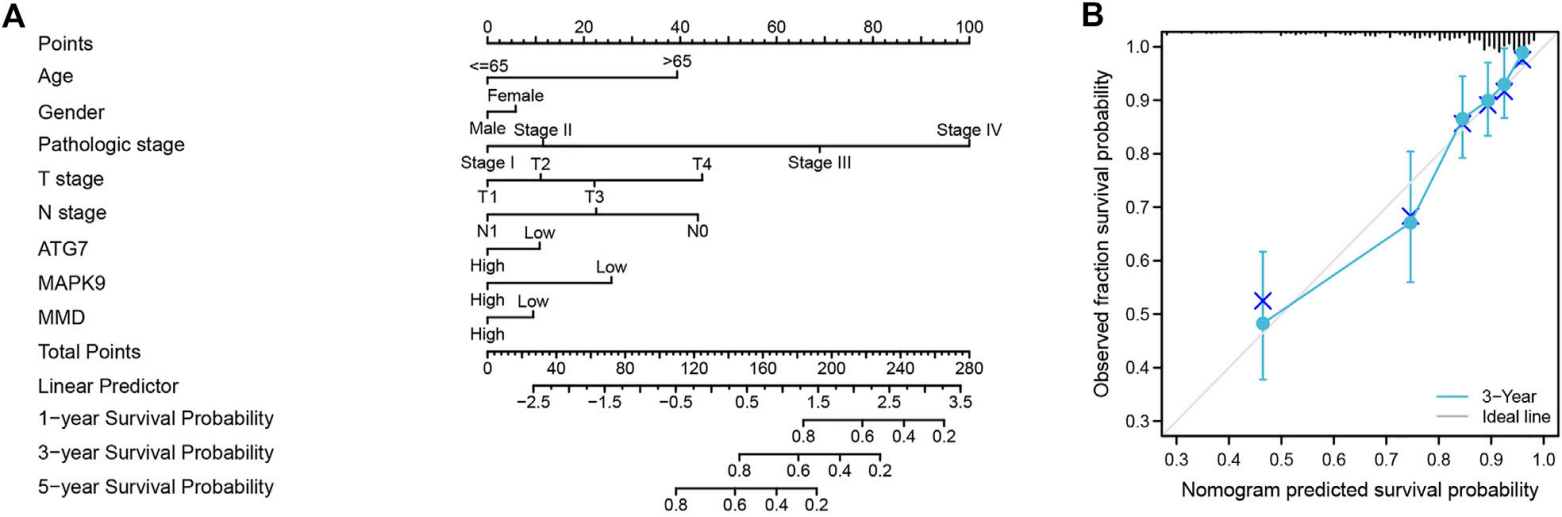
The ferroptosis-related prognostic nomogram and calibration curve. **(A)** A predictive ferroptosis-related prognostic nomogram predicted the 1-year, 3-years, and 5-years OS. **(B)** A calibration curve for the probability of 3-years OS.

Finally, we used the results of the multivariate analysis to establish a predictive ferroptosis-related prognostic nomogram. We used nomogram to predict the 1-year, 2-years, and 3-years OS for identifying the predictive value of age, gender, pathologic stage, TNM stage, and risk score (Figure 6A). This nomogram was used to evaluate the variables, which were based on patients’ characteristics, including age, gender, TNM stage and risk score. The predictive accuracy of OS can be judged by the calibrated curves. As shown in Figure 6B, the calibration curve for the predictive probabilities displayed an accordant agreement for the 3-years OS.

## Discussion

At the moment, surgical resection, chemotherapy, targeted therapy and immunotherapy are the main clinical treatments for CRC (Messersmith 2019; Modest et al., 2019; Biller and Schrag 2021). The option depends on the physical state and clinical characteristics of patients and the tumor stage (Benitez Majano et al., 2019; Roque-Castellano et al., 2020). Unfortunately, all these approaches are not ideal for patients with advanced CRC (Kim 2015). One choice of optimize the clinical treatment scheme is to explore more effective prognostic markers or therapeutic targets. Ferroptosis, a newly discovered type of cell death, is found to function in many cancers and is thought to have great potential for anti-tumor therapy (Tang et al., 2020; Wang et al., 2020). Recently, there are several researches have reported that ferroptosis activation could be benefit for tumor outcomes (Hassannia et al., 2019). For example, wang et al. (Wang et al., 2019) reported that cell ferroptosis is regulated by CD8^+^ T cells, which can in turn affect the efficacy of tumor immunotherapy. Therefore, further study of ferroptosis-related genes in CRC may help to determine the tumor prognosis. Moreover, it is meaningful, to some degree, to make it clearer about the relationship between ferroptosis and tumor immune microenvironment, which can guide for the immunotherapy of CRC.

In our study, we focused on ferroptosis-related genes and their effect on prognosis and sought to explore the relationship between these genes and tumor immune microenvironment. First, we analyzed the DEGs in GEO: GSE21510, GSE44861, GSE62321 and GSE79793 datasets, and intersected them with ferroptosis gene set in FerrDb database. We identified 93 ferroptosis-related genes in CRC this time. Functional analysis of these genes revealed that they were involved in immune system processes and may play roles in the immune microenvironment. Univariate Cox and multivariate Cox regression analysis were used to identify the most significant prognostic genes, which were ATG7, MAPK9 and MMD. Then, the risk score was calculated and the prognostic model was established, which was verified in GEO: GSE41258. Next, we found that these three ferroptosis-related prognostic genes were closely related to immune cells by using the TIMER 2.0 database. CIBERSORT algorithm was performed to evaluate the immune cell infiltration of the tumor immune microenvironment.

In recent years, there have been several bioinformatic studies about tumor associated ferroptosis-related genes in CRC. Moreover, some of them also explored the relationship between ferroptosis-related genes and immune microenvironment. Pan et al. reported a ferroptosis-related prognostic model based on eight genes, AKR1C1, ALOX12, ATP5MC3, CARS1, HMGCR, CRYAB, FDFT1, and PHKG2. They divided the patients in two groups according to the expression pattern of these genes and CD8^+^ T cells were significantly different in the two groups. They though it could be a biomarker for immune checkpoint therapy in CRC Patients (Yang and Zhou 2021). Zheng et al. established a prognostic risk signature based on 10 ferroptosis-related genes, ATG7, PGD, ATP6V1G2, DRD4, DUOX1, JDP2, NOX4, SLC2A3, TP63 and VEGFA. There were different immune landscapes between high and low risk groups (Yang et al., 2021). Besides, He et al. demonstrated that aberrantly expressed MT1G also affected the immune response of CRC patients (Peng et al., 2022). Immune escape is an important reason for tumor treatment failure and the change of tumor environment caused by ferroptosis may be responsible for it (Xu et al., 2020; Wang et al., 2021). According to our results, the ATG7, MAPK9 and MMD were independent ferroptosis-related genes that can used as prognosis markers. Furthermore, they may also be used as biomarkers to predict the response of immunotherapy and as one reference of determining CRC therapeutic strategy. This find further strengthened the understanding that there is a close connection between ferroptosis and T cell immunity or cancer immunotherapy. This can further verify and improve the ferroptosis-related gene map currently and provide clinical potential from another point of view, which may benefit for the imunotherapy and prognosis of CRC in the future.

ATG7, an important member in the autophagy process, participates in autophagosome formation and maturation (Collier and Suomi 2021). It also plays important roles in cancer. It was reported that knockout of ATG7 could lead to a significant reduction in tumorigenesis in a mouse model of hepatocellular carcinoma (Cho et al., 2021). Besides, ATG7 can contribute to the survival of dormant breast cancer cells and metastatic tumor recurrence by activating autophagy (Vera-Ramirez et al., 2018). In addition, autophagy has been shown to promote the ferroptosis process through the ferritin pathway, which is naturally regulated by ATG7 (Hou et al., 2016). The protein encoded by MAPK9 belong to the MAP kinase family and is involved in cell proliferation, differentiation, transcriptional regulation as well as other processes (Li et al., 2017). MAPK9 is most closely related to MAPK8, and both are known as C-Jun N-terminal kinase, which can block tumor suppressor p53 (Topisirovic et al., 2009; Barutcu and Girnius 2018). MAPK9 signaling pathway is reported to have regulatory roles in non-small cell lung cancer, bladder cancer, cholangiocarcinoma and other cancers (Hao et al., 2021; Huang et al., 2021; Teng et al., 2021). Meanwhile, it also has a great potential as the biomarker (Song et al., 2021). What’s more, MAPK9 also functions in many immune-related diseases, suggesting that we could explore its role in the immune environment (Won et al., 2016; Naderi et al., 2019). MMD, first discovered in 1995, is named for its high expression in mature macrophages but not in monocytes (Rehli et al., 1995). Preferentially expressed in mature macrophages, MMD participates in the activation of macrophages with a carcinogenic role (Liu et al., 2012; Lin et al., 2020). There was a research that highlighted the value of miR-140–5p/MMD axis in non-small cell lung cancer (Li and He 2014). In this study, we also explored the significance of MMD in tumor immune microenvironment, which drawed no attention previously.

In conclusion, our study established a prognostic model of CRC based on ferroptosis-related genes ATG7, MAPK9, and MMD and further verified its good predictive effect. The establishment of this model and related immune analysis confirmed the influence of immune microenvironment on the prognosis of CRC, which may benefit for clinical diagnosis and outcome judgment of CRC in the future.

## Data Availability

Publicly available datasets were analyzed in our study. This data can be found from the GEO database (https://www.ncbi.nlm.nih.gov/geo), and TCGA database (https://www.cancer.gov/tcga/).

## Abbreviations

CRC, colorectal cancer; GEO, Gene Expression Omnibus; TCGA, the Cancer Genome Atlas; DEGs, differentially expressed genes; ROS, reactive oxygen species; GO, gene ontology; PPI, protein-protein interaction; ROC, receiver operating characteristic; AUC, area under curve; CC, cellular components; MF, molecular functions; KEGG, Kyoto Encyclopedia of Genes; TIMER, Tumor Immune Estimation Resource.

## References

[B1] Arnold M. Sierra M. S. Laversanne M. Soerjomataram I. Jemal A. Bray F. (2017). Global patterns and trends in colorectal cancer incidence and mortality. Gut 66 (4), 683–691. 10.1136/gutjnl-2015-310912 26818619

[B2] Barutcu S. A. Girnius N. Vernia S. Davis R. J. (2018). Role of the MAPK/cJun NH(2)-terminal kinase signaling pathway in starvation-induced autophagy. Autophagy 14 (9), 1586–1595. 10.1080/15548627.2018.1466013 29950132 PMC6135577

[B3] Benitez Majano S. Di Girolamo C. Rachet B. Maringe C. Guren M. G. Glimelius B. (2019). Surgical treatment and survival from colorectal cancer in Denmark, england, Norway, and Sweden: A population-based study. Lancet. Oncol. 20 (1), 74–87. 10.1016/s1470-2045(18)30646-6 30545752 PMC6318222

[B4] Biller L. H. Schrag D. (2021). Diagnosis and treatment of metastatic colorectal cancer: A review. Jama 325 (7), 669–685. 10.1001/jama.2021.0106 33591350

[B5] Brenner H. Kloor M. Pox C. P. (2014). Colorectal cancer. Lancet 383 (9927), 1490–1502. 10.1016/s0140-6736(13)61649-9 24225001

[B6] Chen H. Li N. Ren J. Feng X. Lyu Z. Wei L. (2019). Participation and yield of a population-based colorectal cancer screening programme in China. Gut 68 (8), 1450–1457. 10.1136/gutjnl-2018-317124 30377193

[B7] Chen X. Kang R. Kroemer G. Tang D. (2021). Ferroptosis in infection, inflammation, and immunity. J. Exp. Med. 218 (6), e20210518. 10.1084/jem.20210518 33978684 PMC8126980

[B8] Cho K. J. Shin S. Y. Moon H. Kim B. K. Ro S. W. (2021). Knockdown of Atg7 suppresses Tumorigenesis in a murine model of liver cancer. Transl. Oncol. 14 (9), 101158. 10.1016/j.tranon.2021.101158 34174688 PMC8243000

[B9] Collier J. J. Suomi F. Olahova M. McWilliams T. G. Taylor R. W. (2021). Emerging roles of ATG7 in human health and disease. EMBO Mol. Med. 13 (12), e14824. 10.15252/emmm.202114824 34725936 PMC8649875

[B10] Dekker E. Tanis P. J. Vleugels J. L. A. Kasi P. M. Wallace M. B. (2019). Colorectal cancer. Lancet 394 (10207), 1467–1480. 10.1016/s0140-6736(19)32319-0 31631858

[B11] Dixon S. J. Lemberg K. M. Lamprecht M. R. Skouta R. Zaitsev E. M. Gleason C. E. (2012). Ferroptosis: An iron-dependent form of nonapoptotic cell death. Cell 149 (5), 1060–1072. 10.1016/j.cell.2012.03.042 22632970 PMC3367386

[B12] Fearnhead H. O. Vandenabeele P. Vanden Berghe T. (2017). How do we fit ferroptosis in the family of regulated cell death? Cell Death Differ. 24 (12), 1991–1998. 10.1038/cdd.2017.149 28984871 PMC5686356

[B13] Hao J. Deng H. Yang Y. Chen L. Wu Q. Yao P. (2021). Downregulation of MCM8 expression restrains the malignant progression of cholangiocarcinoma. Oncol. Rep. 46 (5), 235. 10.3892/or.2021.8186 34523691 PMC8453687

[B14] Hassannia B. Vandenabeele P. Vanden Berghe T. (2019). Targeting ferroptosis to iron out cancer. Cancer Cell 35 (6), 830–849. 10.1016/j.ccell.2019.04.002 31105042

[B15] Hou W. Xie Y. Song X. Sun X. Lotze M. T. Zeh H. J. 3rd (2016). Autophagy promotes ferroptosis by degradation of ferritin. Autophagy 12 (8), 1425–1428. 10.1080/15548627.2016.1187366 27245739 PMC4968231

[B16] Huang H. Fan X. Qiao Y. Yang M. Ji Z. (2021). Knockdown of KNTC1 inhibits the proliferation, migration and tumorigenesis of human bladder cancer cells and induces apoptosis. Crit. Rev. Eukaryot. Gene Expr. 31 (1), 49–60. 10.1615/CritRevEukaryotGeneExpr.2021037301 33639055

[B17] Jiang X. Stockwell B. R. Conrad M. (2021). Ferroptosis: Mechanisms, biology and role in disease. Nat. Rev. Mol. Cell Biol. 22 (4), 266–282. 10.1038/s41580-020-00324-8 33495651 PMC8142022

[B18] Johdi N. A. Sukor N. F. (2020). Colorectal cancer immunotherapy: Options and strategies. Front. Immunol. 11, 1624. 10.3389/fimmu.2020.01624 33042104 PMC7530194

[B19] Kim J. H. (2015). Chemotherapy for colorectal cancer in the elderly. World J. Gastroenterol. 21 (17), 5158–5166. 10.3748/wjg.v21.i17.5158 25954089 PMC4419056

[B20] Koppula P. Zhuang L. Gan B. (2021). Cystine transporter slc7a11/xCT in cancer: Ferroptosis, nutrient dependency, and cancer therapy. Protein Cell 12 (8), 599–620. 10.1007/s13238-020-00789-5 33000412 PMC8310547

[B21] Ladabaum U. Dominitz J. A. Kahi C. Schoen R. E. (2020). Strategies for colorectal cancer screening. Gastroenterology 158 (2), 418–432. 10.1053/j.gastro.2019.06.043 31394083

[B22] Li J. Yao W. Zhang L. Bao L. Chen H. Wang D. (2017). Genome-wide DNA methylation analysis in lung fibroblasts co-cultured with silica-exposed alveolar macrophages. Respir. Res. 18 (1), 91. 10.1186/s12931-017-0576-z 28499430 PMC5429546

[B23] Li J. Cao F. Yin H. L. Huang Z. J. Lin Z. T. Mao N. (2020). Ferroptosis: Past, present and future. Cell Death Dis. 11 (2), 88. 10.1038/s41419-020-2298-2 32015325 PMC6997353

[B24] Li N. Lu B. Luo C. Cai J. Lu M. Zhang Y. (2021). Incidence, mortality, survival, risk factor and screening of colorectal cancer: A comparison among China, europe, and northern America. Cancer Lett. 522, 255–268. 10.1016/j.canlet.2021.09.034 34563640

[B25] Li W. He F. (2014). Monocyte to macrophage differentiation-associated (MMD) targeted by miR-140-5p regulates tumor growth in non-small cell lung cancer. Biochem. Biophys. Res. Commun. 450 (1), 844–850. 10.1016/j.bbrc.2014.06.075 24971538

[B26] Liang C. Zhang X. Yang M. Dong X. (2019). Recent progress in ferroptosis inducers for cancer therapy. Adv. Mat. 31 (51), e1904197. 10.1002/adma.201904197 31595562

[B27] Lin W. Zhou L. Liu M. Zhang D. Yan Y. Chang Y. F. (2020). gga-miR-200b-3p promotes macrophage activation and differentiation via targeting monocyte to macrophage differentiation-associated in HD11 cells. Front. Immunol. 11, 563143. 10.3389/fimmu.2020.563143 33101281 PMC7555432

[B28] Liu Q. Zheng J. Yin D. D. Xiang J. He F. Wang Y. C. (2012). Monocyte to macrophage differentiation-associated (MMD) positively regulates ERK and Akt activation and TNF-α and NO production in macrophages. Mol. Biol. Rep. 39 (5), 5643–5650. 10.1007/s11033-011-1370-5 22203480

[B29] Lu T. Xu R. Li Q. Zhao J. Y. Peng B. Zhang H. (2021). Systematic profiling of ferroptosis gene signatures predicts prognostic factors in esophageal squamous cell carcinoma. Mol. Ther. Oncolytics 21, 134–143. 10.1016/j.omto.2021.02.011 33981829 PMC8080401

[B30] Messersmith W. A. (2019). NCCN guidelines updates: Management of metastatic colorectal cancer. J. Natl. Compr. Canc. Netw. 17 (5.5), 599–601. 10.6004/jnccn.2019.5014 31117039

[B31] Modest D. P. Pant S. Sartore-Bianchi A. (2019). Treatment sequencing in metastatic colorectal cancer. Eur. J. Cancer 109, 70–83. 10.1016/j.ejca.2018.12.019 30690295

[B32] Mohamed A. A. Omar A. A. A. El-Awady R. R. Hassan S. M. A. Eitah W. M. S. Ahmed R. (2020). MiR-155 and MiR-665 role as potential non-invasive biomarkers for hepatocellular carcinoma in Egyptian patients with chronic hepatitis C virus infection. J. Transl. Int. Med. 8 (1), 32–40. 10.2478/jtim-2020-0006 32435610 PMC7227164

[B33] Mou Y. Wang J. Wu J. He D. Zhang C. Duan C. (2019). Ferroptosis, a new form of cell death: Opportunities and challenges in cancer. J. Hematol. Oncol. 12 (1), 34. 10.1186/s13045-019-0720-y 30925886 PMC6441206

[B34] Naderi N. Yousefi H. Mollazadeh S. Seyed Mikaeili A. Keshavarz Norouzpour M. Jazebi M. (2019). Inflammatory and immune response genes: A genetic analysis of inhibitor development in Iranian hemophilia A patients. Pediatr. Hematol. Oncol. 36 (1), 28–39. 10.1080/08880018.2019.1585503 30888230

[B35] Peng B. Peng J. Kang F. Zhang W. Peng E. He Q. (2022). Ferroptosis-related gene MT1G as a novel biomarker correlated with prognosis and immune infiltration in colorectal cancer. Front. Cell Dev. Biol. 10, 881447. 10.3389/fcell.2022.881447 35517502 PMC9065264

[B36] Piawah S. Venook A. P. (2019). Targeted therapy for colorectal cancer metastases: A review of current methods of molecularly targeted therapy and the use of tumor biomarkers in the treatment of metastatic colorectal cancer. Cancer 125 (23), 4139–4147. 10.1002/cncr.32163 31433498

[B37] Rehli M. Krause S. W. Schwarzfischer L. Kreutz M. Andreesen R. (1995). Molecular cloning of a novel macrophage maturation-associated transcript encoding a protein with several potential transmembrane domains. Biochem. Biophys. Res. Commun. 217 (2), 661–667. 10.1006/bbrc.1995.2825 7503749

[B38] Rezapour A. Nargesi S. Mezginejad F. Rashki Kemmak A. Bagherzadeh R. (2021). The economic burden of cancer in Iran during 1995-2019: A systematic review. Iran. J. Public Health 50 (1), 35–45. 10.18502/ijph.v50i1.5070 34178762 PMC8213609

[B39] Roque-Castellano C. Fariña-Castro R. Nogués-Ramia E. M. Artiles-Armas M. Marchena-Gómez J. (2020). Colorectal cancer surgery in selected nonagenarians is relatively safe and it is associated with a good long-term survival: An observational study. World J. Surg. Oncol. 18 (1), 120. 10.1186/s12957-020-01895-8 32493351 PMC7271489

[B40] Simon K. (2016). Colorectal cancer development and advances in screening. Clin. Interv. Aging 11, 967–976. 10.2147/cia.s109285 27486317 PMC4958365

[B41] Song J. Liu Y. Guan X. Zhang X. Yu W. Li Q. (2021). A novel ferroptosis-related biomarker signature to predict overall survival of esophageal squamous cell carcinoma. Front. Mol. Biosci. 8, 675193. 10.3389/fmolb.2021.675193 34291083 PMC8287967

[B42] Stockwell B. R. Jiang X. (2019). A physiological function for ferroptosis in tumor suppression by the immune system. Cell Metab. 30 (1), 14–15. 10.1016/j.cmet.2019.06.012 31269423 PMC6944065

[B43] Sung H. Ferlay J. Siegel R. L. Laversanne M. Soerjomataram I. Jemal A. (2021). Global cancer statistics 2020: GLOBOCAN estimates of incidence and mortality worldwide for 36 cancers in 185 countries. Ca. Cancer J. Clin. 71 (3), 209–249. 10.3322/caac.21660 33538338

[B44] Tang R. Xu J. Zhang B. Liu J. Liang C. Hua J. (2020). Ferroptosis, necroptosis, and pyroptosis in anticancer immunity. J. Hematol. Oncol. 13 (1), 110. 10.1186/s13045-020-00946-7 32778143 PMC7418434

[B45] Teng Z. Yao J. Zhu L. Zhao L. Chen G. (2021). ZNF655 is involved in development and progression of non-small-cell lung cancer. Life Sci. 280, 119727. 10.1016/j.lfs.2021.119727 34144060

[B46] Topisirovic I. Gutierrez G. J. Chen M. Appella E. Borden K. L. Ronai Z. A. (2009). Control of p53 multimerization by Ubc13 is JNK-regulated. Proc. Natl. Acad. Sci. U. S. A. 106 (31), 12676–12681. 10.1073/pnas.0900596106 19651615 PMC2722327

[B47] Vera-Ramirez L. Vodnala S. K. Nini R. Hunter K. W. Green J. E. (2018). Autophagy promotes the survival of dormant breast cancer cells and metastatic tumour recurrence. Nat. Commun. 9 (1), 1944. 10.1038/s41467-018-04070-6 29789598 PMC5964069

[B48] Wang W. Green M. Choi J. E. Gijón M. Kennedy P. D. Johnson J. K. (2019). CD8(+) T cells regulate tumour ferroptosis during cancer immunotherapy. Nature 569 (7755), 270–274. 10.1038/s41586-019-1170-y 31043744 PMC6533917

[B49] Wang Y. Wei Z. Pan K. Li J. Chen Q. (2020). The function and mechanism of ferroptosis in cancer. Apoptosis 25 (11-12), 786–798. 10.1007/s10495-020-01638-w 32944829

[B50] Wang Y. Hou K. Jin Y. Bao B. Tang S. Qi J. (2021). Lung adenocarcinoma-specific three-integrin signature contributes to poor outcomes by metastasis and immune escape pathways. J. Transl. Int. Med. 9 (4), 249–263. 10.2478/jtim-2021-0046 35136724 PMC8802404

[B51] Won Y. H. Lee M. Y. Choi Y. C. Ha Y. Kim H. Kim D. Y. (2016). Elucidation of relevant neuroinflammation mechanisms using gene expression profiling in patients with amyotrophic lateral sclerosis. PLoS One 11 (11), e0165290. 10.1371/journal.pone.0165290 27812125 PMC5094695

[B52] Wu X. Li Y. Zhang S. Zhou X. (2021). Ferroptosis as a novel therapeutic target for cardiovascular disease. Theranostics 11 (7), 3052–3059. 10.7150/thno.54113 33537073 PMC7847684

[B53] Xie Y. Hou W. Song X. Yu Y. Huang J. Sun X. (2016). Ferroptosis: Process and function. Cell Death Differ. 23 (3), 369–379. 10.1038/cdd.2015.158 26794443 PMC5072448

[B54] Xu J. Zhang J. Wang J. (2020). The application of traditional Chinese medicine against the tumor immune escape. J. Transl. Int. Med. 8 (4), 203–204. 10.2478/jtim-2020-0032 33511046 PMC7805287

[B55] Yang C. Huang S. Cao F. Zheng Y. (2021). Role of ferroptosis-related genes in prognostic prediction and tumor immune microenvironment in colorectal carcinoma. PeerJ 9, e11745. 10.7717/peerj.11745 34316400 PMC8286063

[B56] Yang Y. B. Zhou J. X. Qiu S. H. He J. S. Pan J. H. Pan Y. L. (2021). Identification of a novel ferroptosis-related gene prediction model for clinical prognosis and immunotherapy of colorectal cancer. Dis. Markers 2021, 4846683. 10.1155/2021/4846683 34868393 PMC8635899

[B57] Zhou J. Zheng R. Zhang S. Zeng H. Wang S. Chen R. (2021). Colorectal cancer burden and trends: Comparison between China and major burden countries in the world. Chin. J. Cancer Res. 33 (1), 1–10. 10.21147/j.issn.1000-9604.2021.01.01 33707923 PMC7941684

