# Supplementary figures and images for: Screening of ferroptosis-related genes with prognostic effect in colorectal cancer by bioinformatic analysis
Source: Front Mol Biosci. 2022 Sep 20;9:979854. doi: 10.3389/fmolb.2022.979854 (PMC9531163; doi:10.3389/fmolb.2022.979854)

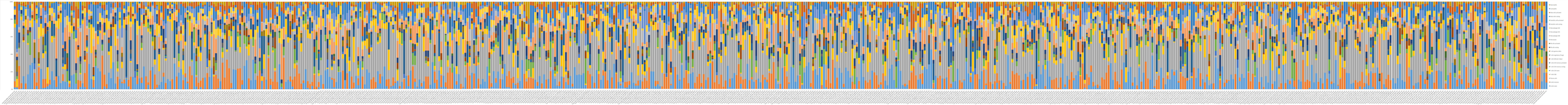

Supplement: Supplementary file 1 [file Image3.JPEG]

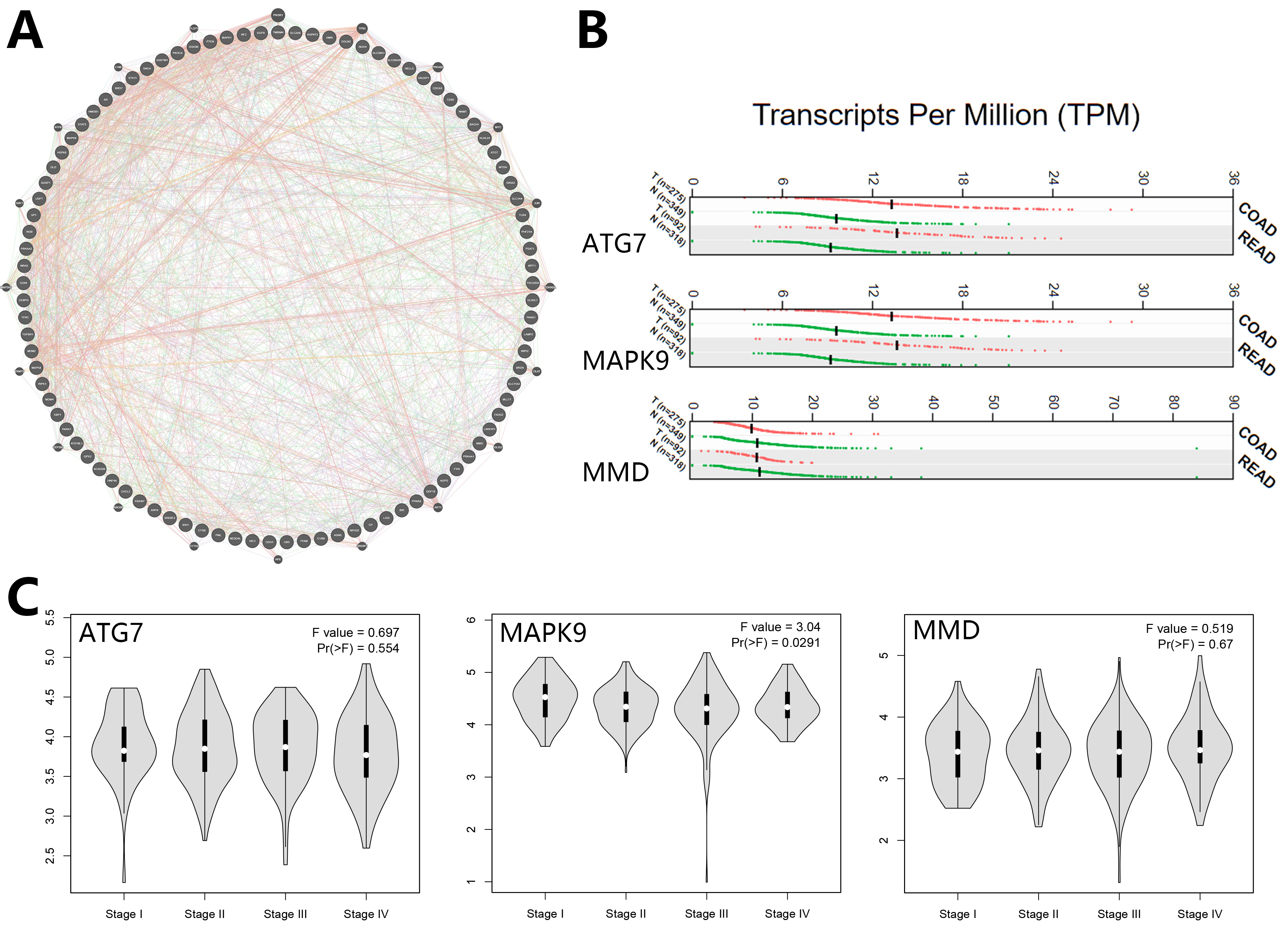

Supplement: Supplementary file 2 [file Image1.JPEG]

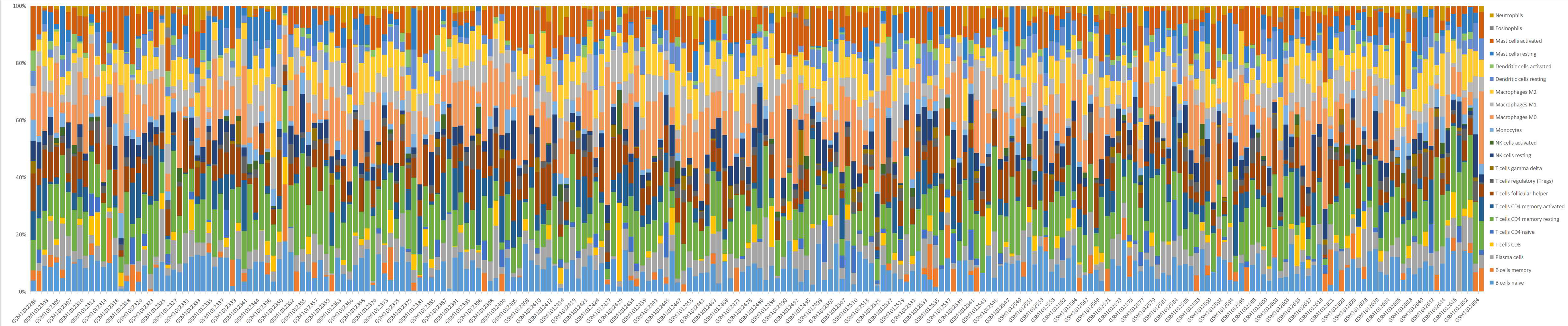

Supplement: Supplementary file 3 [file Image4.JPEG]

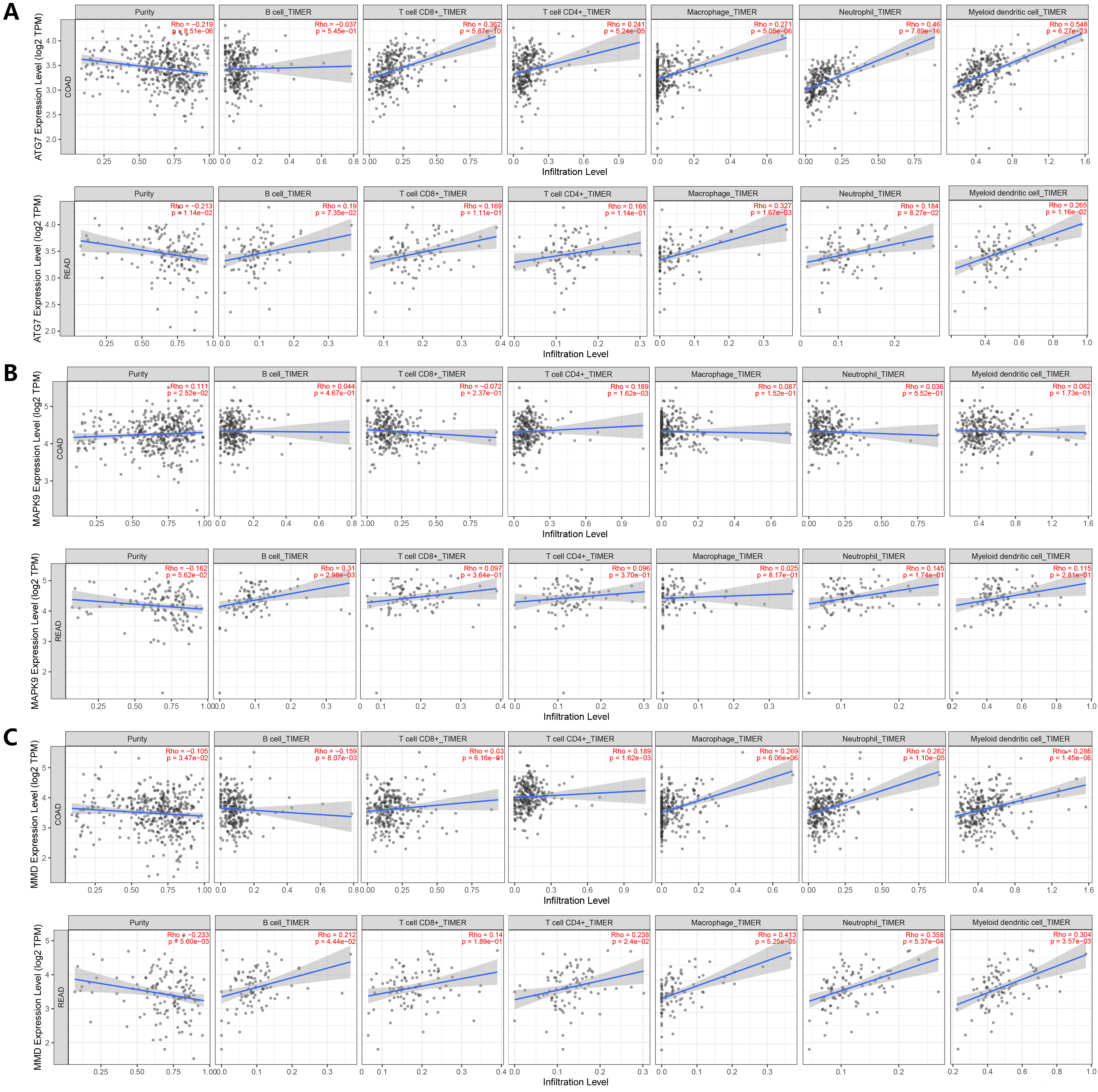

Supplement: Supplementary file 4 [file Image2.JPEG]
